# Changes in diversity and composition of rhizosphere bacterial community during natural restoration stages in antimony mine

**DOI:** 10.7717/peerj.12302

**Published:** 2021-10-14

**Authors:** Renyan Duan, Yuxiang Lin, Jianing Zhang, Minyi Huang, Yihuan Du, Li Yang, Jing Bai, Guohong Xiang, Zhigao Wang, Yaqi Zhang

**Affiliations:** 1 College of Agriculture and Biotechnology, Hunan University of Humanities, Science and Technology, Loudi, Hunan, China; 2 Zhejiang Forest Academy, Hangzhou, China

**Keywords:** Heavy metal pollution, Microbial community structure, 16S rRNA, Rhizosphere

## Abstract

**Background:**

Open pit antimony (Sb) mining causes serious soil pollution, and phytoremediation is a low-cost approach to remediate heavy metal contaminated soil. Rhizosphere bacteria play an important role in ecological restoration in mining areas. There is a knowledge gap on how to find suitable rhizosphere microorganisms to improve the phytoremediation effect. Understanding the differences of rhizosphere bacterial diversity in different restoration stages is helpful to find suitable bacteria for ecological restoration.

**Methods:**

A method of the substitution of “space” for “time” was used to study the effect of natural restoration on rhizosphere bacterial community. According to the dominant vegetation types (herb, shrub, and tree) in the natural restoration area of Sb mining, the early restoration (ER), middle restoration (MR), and later restoration (LR) from the largest Sb mine (Xikuangshan mine) in the world were selected to evaluate the differences in the composition and diversity of rhizosphere bacteria during three natural restoration stages. Each restoration stage had five samples. To determine the relationship between restoration stages and bacterial diversity in the rhizosphere, high throughput sequencing of PCR amplified were used.

**Results:**

Alpha diversity, as assessed by Chao indices, appeared lowest in ER but this trend was not seen with other diversity metrics, including the Simpson and Shannon. Beta diversity analysis suggested there were differences in rhizobacterial community structure associate with restoration stage. At the phylum level, natural restoration led to a significant increase in the relative abundance of *Actinobacteria* in the MR, and a significant decrease in the relative abundance of *Patescibacteria* in the LR. Additionally, *Calditrichaeota*, *Deferribacteres* and *Epsilonbacteraeota* were only found in ER. At the genus level, the relative abundance of *RB41* and *Haliangium* were highest in LR plots, while that of *Bacillus* and *Gaiella* were highest in ER plots. Additionally, the *Azorhizobium* genus was only detected in the ER phase. Overall, our findings suggested that several rhizosphere microbial communities had significant differences among three natural restoration stages (ER, MR, and LR) and the rhizosphere bacterial communities mainly appeared in the early restoration stage can be preferred for remediation of pollution soil in Xikuangshan.

## Introduction

Antimony (Sb) is an important trace element in the world economy with an annual production of about 150,000 t (Shtangeeva, Bali & Harris, 2011). It is widely used in the production of ceramics, glasses, batteries, pyrotechnic materials, paints, ammunition, flame retardants, semiconductors, synthetic fabrics, etc. (Wilson et al., 2010). Open pit Sb mining can damage the landscape and vegetation and contaminate soils with high levels of Sb (*e.g.*, Sb(III), Sb(V)) and other heavy metals (*e.g.*, As, Zn, Pb, Cd, and Hg) (Okkenhaug et al., 2011; Yang, He & Wang, 2015; Tang et al., 2019; Zhou, Hursthouse & Chen, 2019). Sb contamination of soils inhibits plant growth (Shtangeeva, Bali & Harris, 2011), influences the stucture, of rhizosphere soil microbial community (Guo et al., 2019), and even causing health risk of teratogenesis and carcinogenesis to human body (WHO, 2003). Given the toxicity and biological harm of Sb, Sb and its compounds have long been listed as priority pollutants by the environmental protection structure of European Union and United States (He et al., 2012; Filella, Belzile & Chen, 2002). To reduce the ecological and health risks in Sb mining areas, it is critical to study appropriate environmental restoration measures for Sb-contaminated soil.

Recently, phytoremediation has been widely used to remediate heavy metal contaminated soil. This low-cost approach has less environmentally impact than chemical remediation technology (Boyd, 2007). However, the success of phytoremediation to pollution soils in metal mining areas depends not only on the selection of heavy metal enrichment-plants (Orozco-Aceves, Tibbett & Standish, 2017; Audino, Louzada & Comita, 2014), but also on the selection of soil microorganism, especially rhizosphere soil microorganism, which are not as well studied (Wei et al., 2015). Rhizosphere microorganisms play an important part in the process of plant-soil ecosystem processes, including nutrient cycling, energy transfer, metal resistance, and detoxification, as well as the establishment of sustainable plant communities (Mishra, Singh & Arora., 2017). Many rhizospheric microorganisms, particularly some plant-growth-promoting rhizosphere bacteria, can increase biomass production and/or decrease the accumulation of heavy metal in plants (Mishra, Singh & Arora., 2017; Lebeau, Braud & Jezequel, 2008). As one of the most abundant microbial groups in soil microorganism, rhizosphere bacteria actively participate in various biogeochemical reactions in rhizosphere and soil (Mishra, Singh & Arora., 2017). Compared to non-rhizosphere bacteria in soil, rhizosphere bacteria have more direct effects on the growth and development of root (Mishra, Singh & Arora., 2017). In addition, rhizosphere bacteria are more active and have high sensitivity to any small changes in environmental stress, which can be used as an early effective biological indicator to evaluate heavy metal pollution and plant growth status (Deng et al., 2015; Muehe et al., 2015). Therefore, the examination of the diversity and community structure of indigenous rhizosphere bacteria under heavy metal stress at different vegetation restoration stages can provide valuable guidance for the remediation of Sb contaminated soil.

However, studies on microorganisms in Sb mine area were mainly focused on soil microorganisms and the arbuscular mycorrhizal fungi of heavy metal hyperaccumulators. For example, Wang et al. (2018) found that the relative abundance and alpha diversity of bacterial communities in soil varied along with Sb-contaminated soil gradients. Another study on the molecular diversity and community composition of arbuscular mycorrhizal fungi in the rhizosphere of three heavy metal hyperaccumulators (*Miscanthus anderss, Boehmeria nivea* and *Cynodon dactylon*) was carried out in an Sb mining area (Wei et al., 2015), but it only described the community structure of arbuscular mycorrhizal fungi in plant rhizosphere in detail, ignoring the rhizosphere bacteria. As we know, only one study investigated the changes of rhizosphere bacterial community during the remediation process of heavy metal enriched plants around Sb mining areas, and found that remediation affected rhizosphere bacterial diversity and most of rhizosphere bacteria belonged to the *Acidobacteria*, *Bacteroidetes*, *Proteobacteria*, and *Actinobacteria* (Guo et al., 2019). At present, some artificial vegetation restoration measures have been carried out in Sb mines, but due to the cost and technical reasons, natural ecological restoration measure is the main method in maintaining ecosystem health (Ding, Yang & Deng, 2013; Nakamaru & Martín Peinado, 2017; Sun, Li & Wu, 2021). Despite widespread recognition of natural restoration importance, little information is available on the diversity and community composition of rhizosphere bacterial communities on Sb contaminated soil during natural vegetation restoration progresses. This knowledge gap may hinder the application of rhizosphere bacteria in the remediation of Sb-contaminated soil.

China has the most abundant Sb productions and reserves with 617 Sb mines in 18 provinces (Ding, Yang & Deng, 2013). Xikuangshan mine (Hunan, China), the largest Sb mining in the world, known as the “the Sb capital of the world,” has about 1.1 million tons in reserve of Sb, accounting for about 80% of the country (Tang et al., 2019). Mining activities over nearly 100 years have caused serious soil pollution in mining areas (Okkenhaug et al., 2011; Mo et al., 2013), and the average content of Sb in soils in Xikuangshan mine was up to 4,368.222 mg/kg, which is far higher than that in other Chinese soil (about 1.06 mg/kg) and in global soil (about one mg/kg) (Tang et al., 2019). The extremely high concentration of Sb makes the Xikuangshan mine an excellent model to study the changes in rhizosphere microbial composition during different natural restoration stages. We hypothesized that vegetation restoration could improve the diversity of rhizosphere microbial community and change the composition of rhizosphere microbial community. The aim of this present work was to observe and compare the changes of composition and diversity in rhizosphere microbial communities during natural restoration. This study is helpful to select the suitable rhizosphere microbial community to accelerate the ecological restoration process in Sb mines.

## Materials and Methods

### Site information

**Figure 1:**
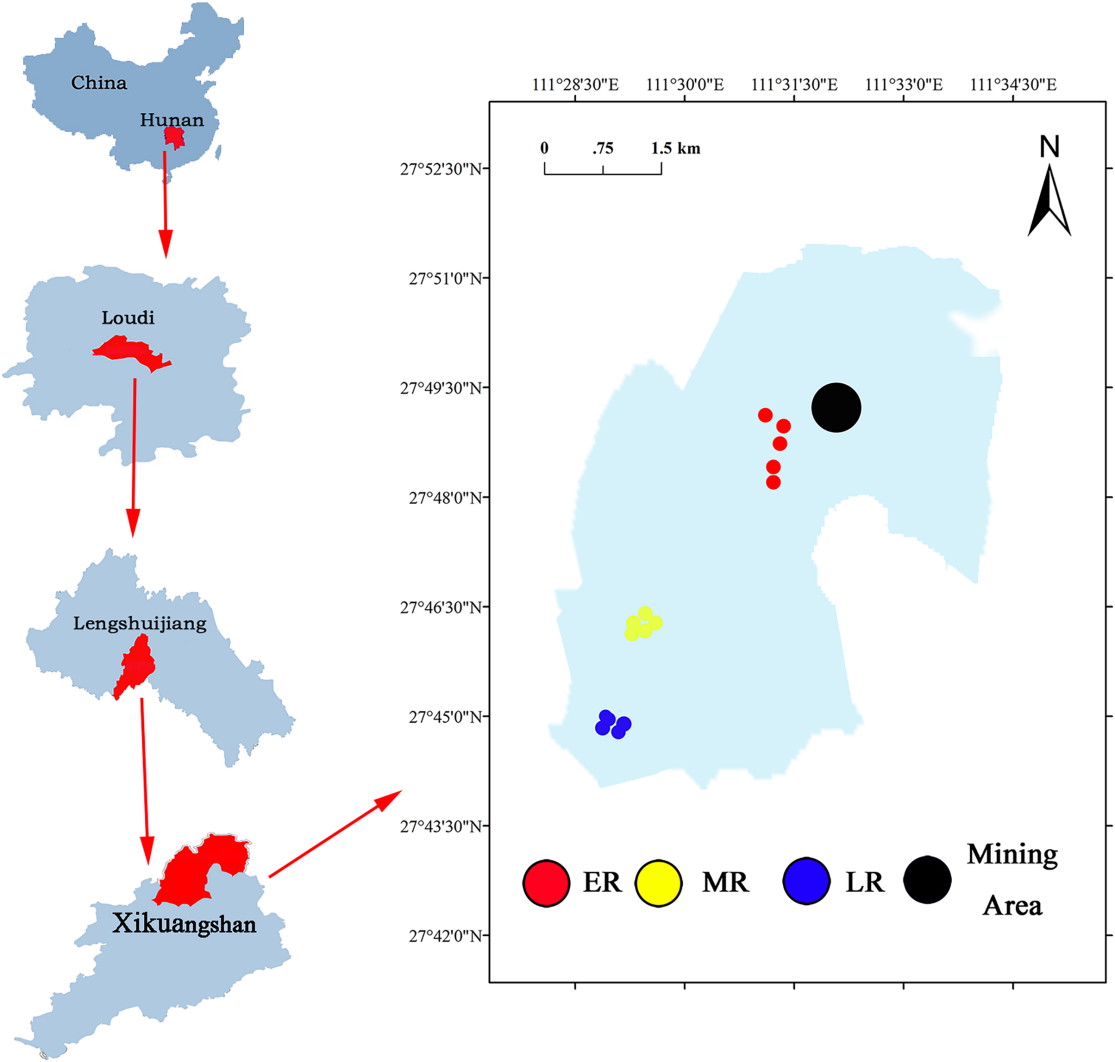
Site location of the study area.

The Xikuangshan Sb mining is located in Lengshuijiang City, Hunan Province, China (111°18′57″–111°36′40″E, 27°30′49″–27°50′38″N) (Fig. 1). The total area is about 439 km^2^. The climate in this area is a subtropical monsoon climate. The annual mean temperature is about 16.7 °C and the annual average precipitation is around 1,381 mm. The minimum temperature is −10.9 °C (January 30, 1977) and the maximum temperature is 39.7 °C (July 26, 1971). The soil type is red soil. After stopping mining, local governments have adopted a series of methods to restore the vegetation and landscape of Sb mining area. However, due to cost and technical reasons, the large areas are still in the stage of natural ecological restoration. The common domain plants in a natural recovery zone mainly include *Pinus massoniana*, *Broussonetia papyrifera*, *Buddleja lindleyana*, *Miscanthus sinensis*, *Conyza canadensis*, *Miscanthus sinensis*, and *Artemisia carvifolia*.

### The collection of rhizosphere soil and determination of heavy metal content

A common and effective method (the substitution of “space” for “time”) of investigating natural succession in the field experiment (Lozano et al., 2014; Cline & Zak, 2015; Zhang et al., 2017) was used to study the effect of natural restoration on rhizosphere bacterial community. Three different recovery phases including early restoration (ER), middle restoration (MR), and later restoration (LR) were selected as our experimental sites in August 2019, according to the dominant vegetation types (herb, shrub, and tree) in the natural restoration area of Sb mining. The characteristics of each stage were presented in Table 1. In each recovery phase, five 1 m × 1 m samples of similar habitat were selected. The dominant plants in the same recovery stage were the same or similar. The dominant plants in ER, MR, and LR were herb (*B. pilosa, C. canadensis*, *M. sinensis* and *A. carvifolia*), shrub (*B. lindleyana*), and tree (*P. massoniana*), respectively.

**Table 1:** Coordinate and dominant plant type (species) of all sampling points.

Recovery phase	Sample	Longitude	Latitude	Altitude (m)	Dominant plant type (species)
ER	ER1	27°48′34.416″E	111°31′23.487″N	662.25	Herb (*Bidens pilosa*, *Miscanthus sinensis*, *Artemisia carvifolia*)
	ER2	27°48′34.294″E	111°31′23.625″N	649.25	Herb (*Conyza canadensis*, *Miscanthus sinensis*, *Artemisia carvifolia*)
	ER3	27°48′41.886″E	111°31′23.815″N	651.11	Herb (*Bidens pilosa*, *Conyza canadensis*, *Miscanthus sinensis*)
	ER4	27°48′41.908″E	111°31′23.905″N	664.72	Herb (*Bidens pilosa*, *Miscanthus sinensis*, *Artemisia carvifolia*)
	ER5	27°48′40.450″E	111°31′24.211″N	662.19	Herb (*Bidens pilosa*, *Conyza canadensis*, *Miscanthus sinensis*)
MR	MR1	27°46′26.908″E	111°29′47.501″N	590.54	Shrub (*Buddleja lindleyana*)
	MR2	27°46′27.152″E	111°29′45.276″N	597.21	Shrub (*Buddleja lindleyana*)
	MR3	27°46′26.634″E	111°29′44.250″N	504.82	Shrub (*Buddleja lindleyana*)
	MR4	27°46′25.925″E	111°29′44.182″N	512.06	Shrub (*Buddleja lindleyana*)
	MR5	27°46′25.885″E	111°29′44.199″N	504.84	Shrub (*Buddleja lindleyana*)
LR	LR1	27°45′18.899″E	111°28′41.495″N	319.25	Tree (*Pinus massoniana*)
	LR2	27°45′17.525″E	111°28′36.800″N	353.14	Tree (*Pinus massoniana*)
	LR3	27°45′16.927″E	111°28′35.670″N	319.82	Tree (*Pinus massoniana*)
	LR4	27°45′17.302″E	111°28′36.415″N	383.02	Tree (*Pinus massoniana*)
	LR5	27°45′17.006″E	111°28′35.544″N	319.25	Tree (*Pinus massoniana*)

Each recovery phase included five soil samples and each soil sample had five rhizosphere soil subsamples. In each subsample, soils adhering to plant roots were collected using a uniform sampling protocol. The fine roots with soil were collected from the subsamples at a depth of 5–10 cm. Fine roots were shaken gently to obtain rhizosphere soil (Kremer, 2019). Five subsamples were mixed to obtain a composite soil sample. Each composite soil sample was packed in sterile polyethylene bags and transported to laboratory with dry ice. Each sample was divided into two subsets. The first subset was air-dried at <35 °C and sieved (two-mm) for measuring heavy metal content (Sb, As, Pb, Cd, Hg and Zn) with PinAAcle 900 Atomic Absorption Spectrometer (PerkinElmer Inc., Waltham, MA, USA). The second subset was stored in sterilized 15 mL centrifuge tubes in foam box filled with dry ice and frozen at −80 °C for genomic DNA extraction.

### Illumina MiSeq platform sequencing analysis

Total soil microbial DNA was purified from 0.6 g tested samples using E.Z.N.A. DNA Kit (Omega Bio-Tek, Norcross, GA, USA). Amplification and sequencing of bacterial 16S rRNA gene were performed using forward primers (338F, 5′-ACTCCTACGGGAGGCAGCAG-3′) and reverse primers (806R, 5′-GGACTACHVGGGTWTCTAAT-3′) (Deng et al., 2020). The PCR thermal cycle included an initial denaturation at 98 °C for 5 min followed by 25 cycle reactions. Each reaction included denaturation at 98 °C for 15 s, annealing at 55 °C for 30 s, extending at 72 °C for 30 s, and finally extending for 5 min at 72 °C. Finally, the high-throughput sequencing of 16S rRNA amplicon was performed on the Illumina MiSeq platform at Shanghai Personal Biotechnology Co., Ltd.

### Biodiversity and statistical analysis

The OTU table in QIIME was used to calculate the alpha diversity index of rhizosphere microbial community, including the Chao richness, Shannon Index, Simpson Index, and Pielou Index. Principal coordinates analysis (PCoA) and MNDS based on Bray-Curtis distance were performed to assess the beta diversity of rhizosphere microbial community among three natural restoration stages (ER, MR, and LR). The PCoA based on QIIME with unweighted UniFrac distance was used to quantify the differences between samples. A Venn diagram of three natural recovery phases (ER, MR, and LR) was created by R software (Fouts et al., 2012). QIIME was performed to map community composition and abundance at different taxonomic levels (Oberauner et al., 2013). The Kruskal Wallis test and one-way ANOVA (SPSS version 23.0) were used to test the significant differences at *p* = 0.05.

## Results

### Heavy metal concentration

The heavy metal concentrations (Sb, As, Pb, Cd, Hg, and Zn) in rhizosphere soil varied remarkably among three natural restoration stages (ER, MR, and LR), as listed in Table 2. After natural restoration, the content of Sb from the ER with 5,959 mg/kg decreased to relatively low content in MR and LR with 839 and 68.7 mg/kg, respectively. There were significant differences in Sb content among the three recovery stages (ER, MR, and LR) (*p* < 0.05). The contents of other associated heavy metals (As, Pb, Cd, Hg, and Zn) were also significantly different among the three recovery stages (*p* < 0.05), which followed the order of ER > MR > LR (Table 2).

**Table 2:** Heavy metal concentration in rhizosphere soil among three natural restoration stages (ER, MR, and LR).

Restoration stage	Sb (mg/kg)	As (mg/kg)	Cd (mg/kg)	Pb (mg/kg)	Zn (mg/kg)	Hg (mg/kg)
ER	5,959.00 ± 702.20 a	78.84 ± 11.55 a	17.38 ± 3.03 a	266.00 ± 62.95 a	722.00 ± 58.31 a	3.00 ± 1.38 a
MR	839.00 ± 89.87 b	38.55 ± 6.24 b	1.27 ± 0.44 b	54.00 ± 8.28 b	115.00 ± 12.93 b	0.75 ± 0.11 b
LR	68.70 ± 1.49 c	10.69 ± 1.35 c	0.78 ± 0.14 c	16.60 ± 3.70 c	108.00 ± 8.01 b	0.18 ± 0.05 b

**Notes:**

Data were reported as Mean ± Standard error (*n* = 5). Different letters in the same column indicate significant differences among different natural restoration stages (ANOVA, Tukey’s test, *p* < 0.05).

ER, Early restoration stage; MR, Middle restoration stage; LR, Later restoration stage.

### DNA sequence data and operational taxonomic unit

**Figure 2:**
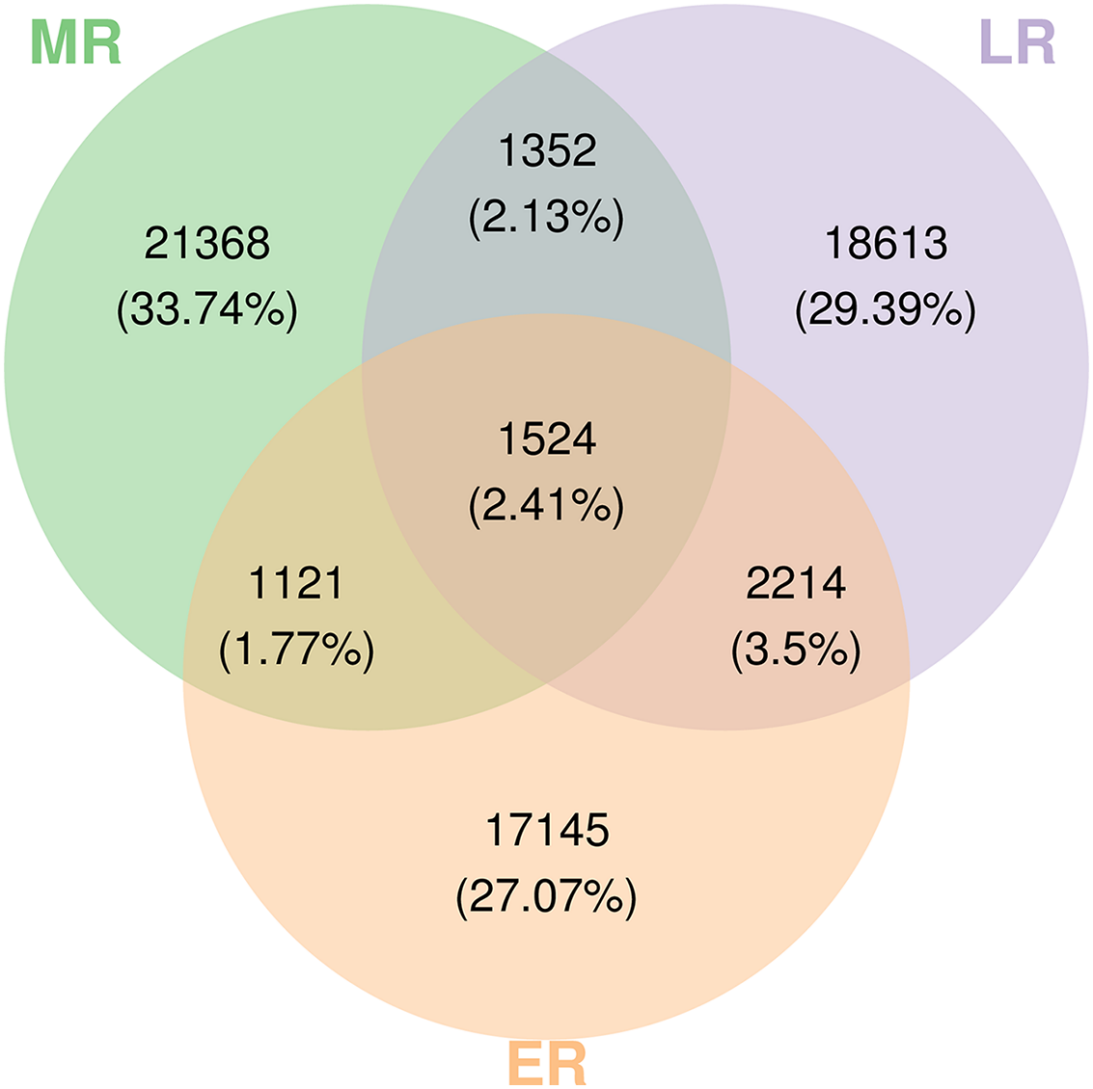
Venn diagrams showing the number of unique and shared OTUs (at 97% identity) in rhizosphere microbial communities among three natural restoration stages (ER, MR, and LR). Different colored shapes represent different groups. ER, Early restoration stage; MR, Middle restoration stage; LR, Later restoration stage.

A total of 3,152,700 raw sequences were gained in the 15 composite rhizosphere soil samples, and 187,325 effective sequences ranging from 127,839 to 272,162 were randomly selected from each sample for fair comparison. The sparse curves of all samples reached a platform, indicating that the data were reasonable and the sequencing depth was sufficient (Fig. S1). These sequences were classified into 63,337 OTUs according to 97% phase sequence similarity comparison. The numbers of identified OTUs in ER, MR, and LR were 22,004, 25,365, and 23,703, respectively (Fig. 2). Among them, there were 1,524 overlapping OTUs in the three groups, and rhizosphere soils from ER, MR, and LR had 17,145, 21,368 and 18,613 unique OTUs, respectively (Fig. 2).

### Alpha and beta diversity analysis

**Figure 3:**
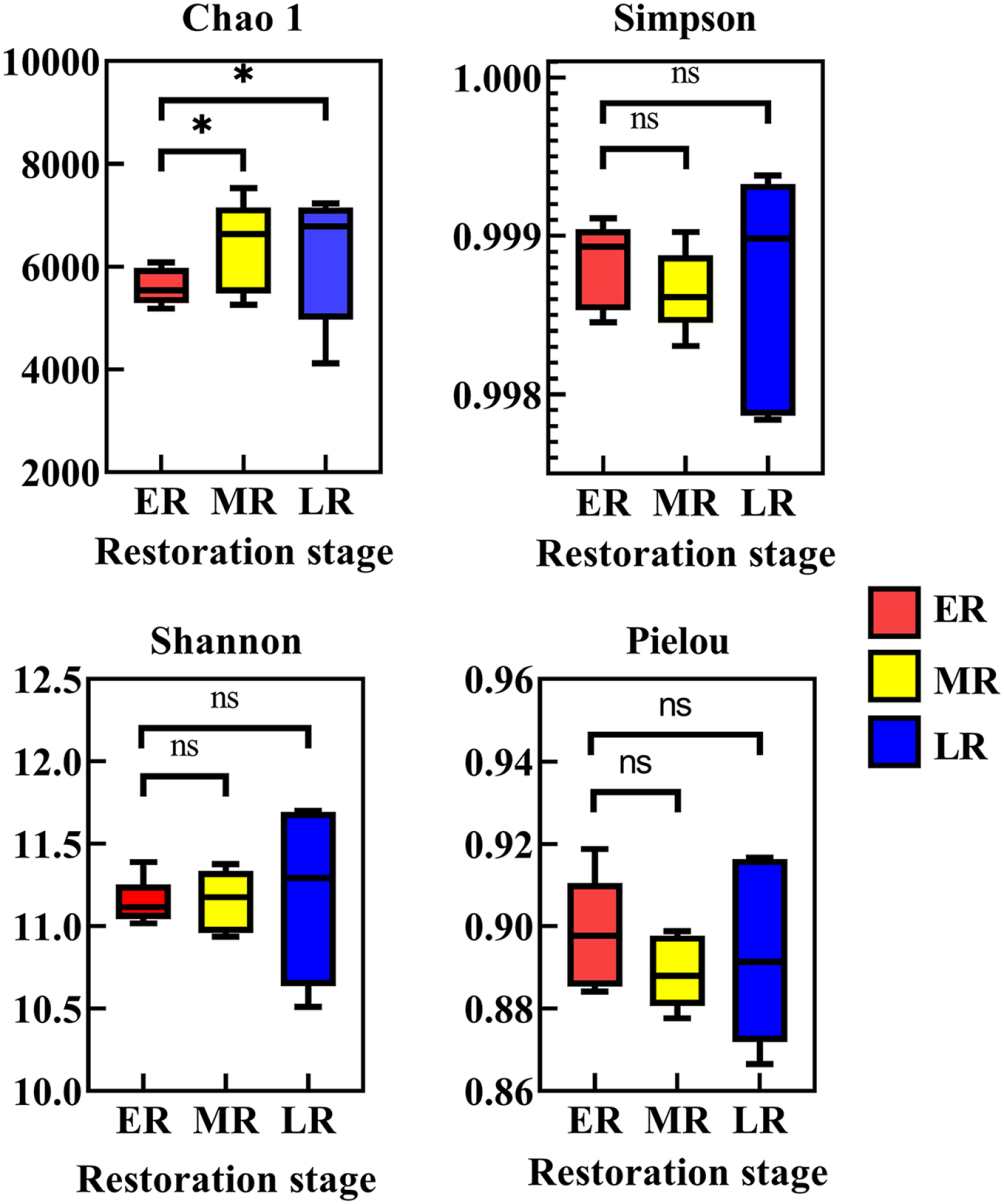
Alpha diversity of rhizosphere microbial communities among three natural restoration stages (ER, MR, and LR). ER, Early restoration stage; MR, Middle restoration stage; LR, Later restoration stage. Different letters indicate significant differences among different natural restoration stages (ANOVA, Tukey’s test, *p* < 0.05). Data are reported as Mean ± Standard error (*n* = 5).

Alpha diversity among three natural restoration stages (ER, MR, and LR) was estimated using the Chao, Simpson, Shannon, and Pielou Indexes. Richness, as assessed by Chao indices, was higher in the MR and LR were significantly higher than that of the ER (*p* < 0.05), while there was no significant difference between the MR and LR (*p* > 0.05) (Fig. 3). The Simpson index, Shannon index and Pielou index all had no significant difference among three natural restoration stages (ER, MR, and LR) (*p* > 0.05) (Fig. 3).

**Figure 4:**
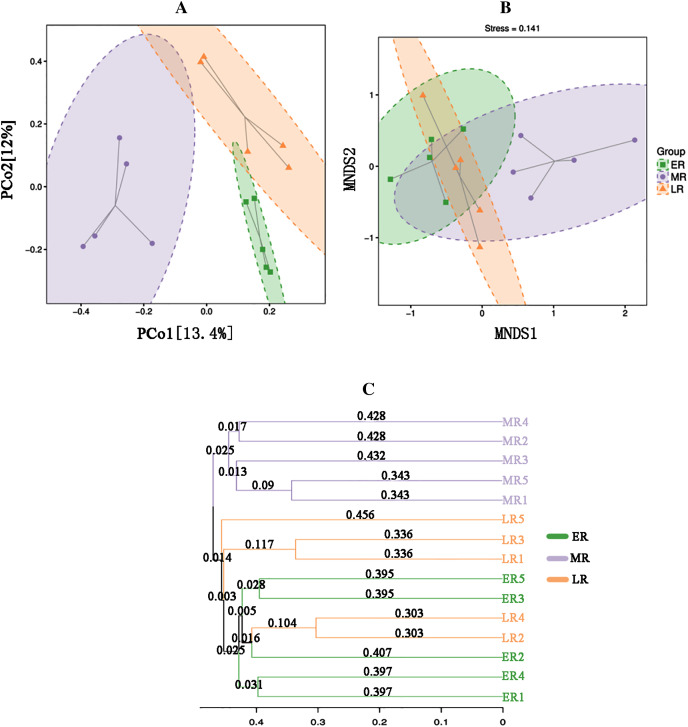
Principal coordinates analysis (PCoA) (A), MNDS (B) and the UPGMA tree (C). Principal Coordinates Analysis (PCoA) (A) and MNDS (B) based on Bray–Curtis distance represents the differences in the rhizosphere bacterial community among three natural restoration stages (ER, MR, and LR). The UPGMA tree (C) showing clusters of bacterial communities based on weighted UniFrac with 100% support at all nodes. Different colored shapes represent different groups. ER, Early restoration stage; MR, Middle restoration stage; LR, Later restoration stage.

Beta diversity analysis showed natural restoration led to changes in the structure of the bacterial community in the rhizosphere. PCoA plot showed that the rhizosphere microbiota of all samples were basically divided into three groups (Fig. 4A). PCoA plot explained 13.4% (PCo1) and 12% (PCo2) of the variation in microbial communities, respectively. In summary, natural restoration led to changes in rhizosphere microbial structure (Fig. 4A). NMDS plot showed that there were some differences among the three restoration stages (Fig. 4B). Furthermore, the UPGMA cluster dendrogram (Fig. 4C) and Permanova and Anosim analysis (Table 3) also showed that there were significant differences in microbial communities among three natural restoration stages (ER, MR and LR).

**Table 3:** Differences of microbial community among three natural restoration stages (ER, MR, and LR).

Treatment		Permanova		Anosim	
Group1	Group2	pseudo-F	*p*-value	R	*p*-value
ER	MR	1.836561	0.014	0.752	0.007
MR	LR	1.930475	0.012	0.648	0.008
LR	ER	1.668150	0.010	0.512	0.007

### Taxonomic composition analysis

**Figure 5:**
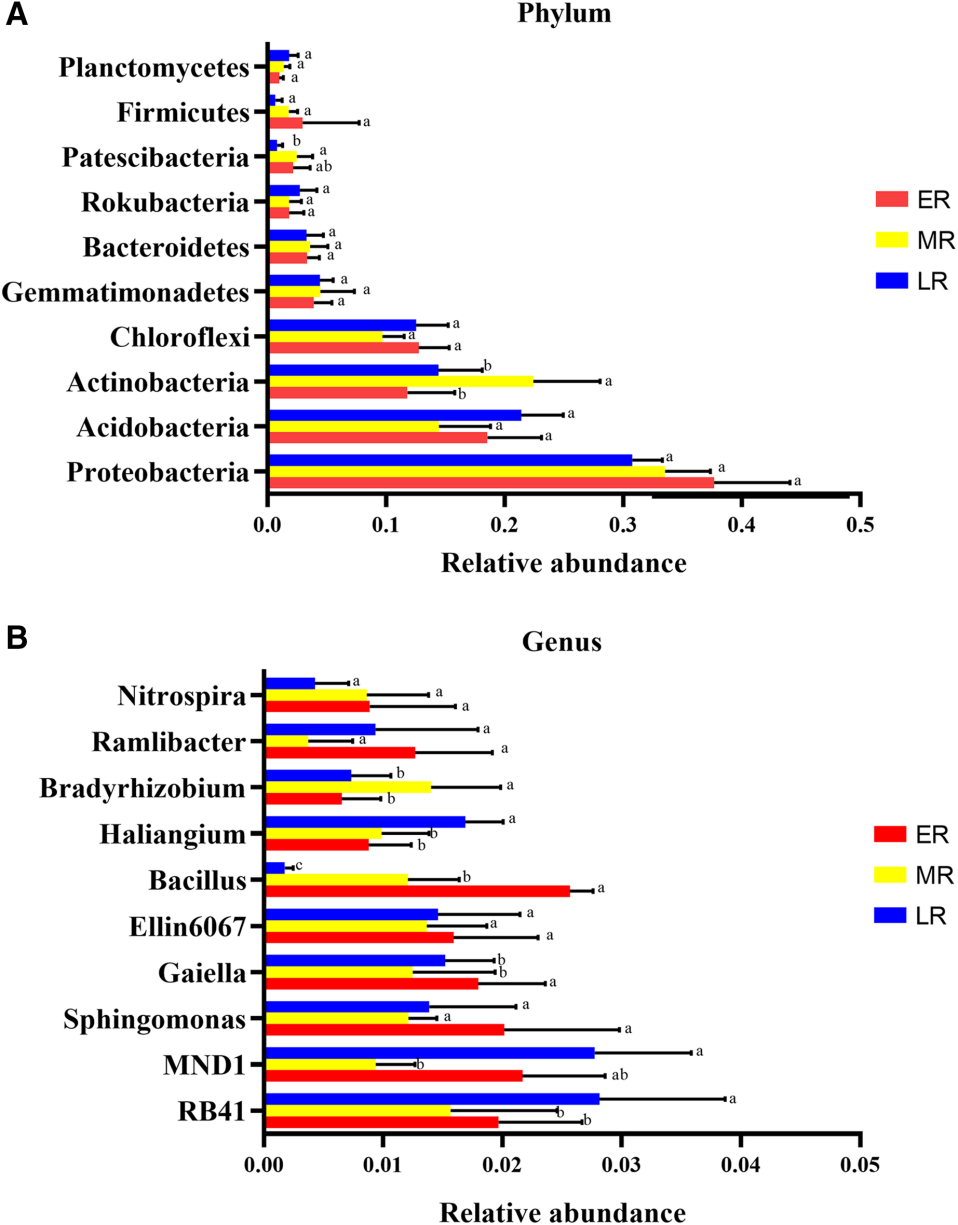
The difference of relative abundance of dominant bacterial phyla and genus among three natural restoration stages. (A) The relative abundance of 10 dominant bacterial phylum (relative abundance > 1% in at least in one sample). (B) The relative abundance of the top 10 bacterial genus. Different letters indicate significant differences among different natural restoration stages (Mean ± SE, Kruskal-Wallis test, *p* < 0.05). ER, Early restoration stage; MR, Middle restoration stage; LR, Later restoration stage.

Microbiota composition analysis showed natural restoration led to changes in the relative abundance in the rhizosphere bacteria. At the phyla level, a total of 40 phylas were detected in this experiment, of which three phylas (*Calditrichaeota*, *Deferribacteres* and *Epsilonbacteraeota*) were only found in the ER stage (Table 4). Ten domain bacterial phyla (the relative abundance > 1%) were *Proteobacteria*, *Acidobacteria*, *Actinobacteria*, *Chloroflexi*, *Gemmatimonadetes*, *Bacteroidetes*, *Rokubacteria*, *Firmicutes*, *Patescibacteria*, and *Planctomycetes* (Fig. 5A). The dominant bacterial phyla with the relative abundance > 10% were *Proteobacteria* (31.5–33.7%), *Acidobacteria* (14.5–20.6%), *Actinobacteria* (11.8–22.4%), and *Chloroflexi* (10.1–15.6%) (Fig. 5A). The proportions of these dominant phyla were different among the three natural restoration stages (Fig. 5A). The abundance of *Proteobacteria*, the most dominant bacteria, were 36.7%, 33.5%, and 31.5% in the ER, MR, and LR, respectively. In addition, the relative abundance of *Actinobacteria* significantly increased in the MR compared to the ER and LR, and that of *Patescibacteria* significantly decreased in the LR compared to the MR (*p* < 0.05, Fig. 5A).

**Table 4:** The phylum and genus (relative abundance of the top 10 bacterial genera) only appeared in the early stage of natural recovery (ER).

Phylum	Relative abundance	Genus	Relative abundance
Calditrichaeota	0.00249	Azorhizobium	0.00307
Epsilonbacteraeota	0.00141	Elioraea	0.000235
Deferribacteres	0.000838	Actinotalea	0.000218
		Aquipuribacter	7.82E−05
		Thermoflexus	4.47E−05
		Falsirhodobacter	4.19E−05
		Ornithinimicrobium	3.35E−05
		Chloroflexus	3.07E−05
		Porphyrobacter	1.68E−05
		Nubsella	1.12E−05

At the genus level, the top ten identified bacterial genera were *RB41*, *MND1*, *Bacillus*, *Ellin6067*, *Gaiella*, *Sphingomonas*, *Haliangium*, *Ramlibacter*, *Nitrospira*, and *Bradyrhizobium* (Fig. 5B). The most dominant genus of the average abundance was *RB41*, which accounted for 1.6%, 1.8%, and 2.8% in the ER, MR, and LR stages, respectively (Fig. 5B). *RB41*, *MND1*, *Bacillus*, *Haliangium*, *Gaiella*, and *Bradyrhizobium* had significant differences in the abundance among three natural restoration stages (*p* < 0.05, Fig. 5B). The average abundance of *RB41* and *Haliangium* were significantly higher in the LR than in the ER and MR. The average abundance of *Bacillus* and *Gaiell*a were significantly lower in the LR than in the ER and MR (*p* < 0.05, Fig. 5B). In addition, the average abundance of *MND*1 (the sub-dominant genus) was the lowest in the MR stage, and the highest in the ER and LR stages. The average abundance of *Bradyrhizobium* was the highest in MR stage, and the lowest in ER and LR stages (*p* < 0.05, Fig. 5B).

Considering the role of rhizobia in promoting plant growth, we focused on the changes of rhizobium in different restoration stages. In this experiment, we detected four rhizobium genera, including *Azorhizobium*, *Allorhizobium-Neorhizobium-Pararhizobium-Rhizobium*, *Mesorhizobium*, and *Bradyrhizobium*. Among this, *Azorhizobium* was only detected in the ER phase (Table 4).

**Figure 6:**
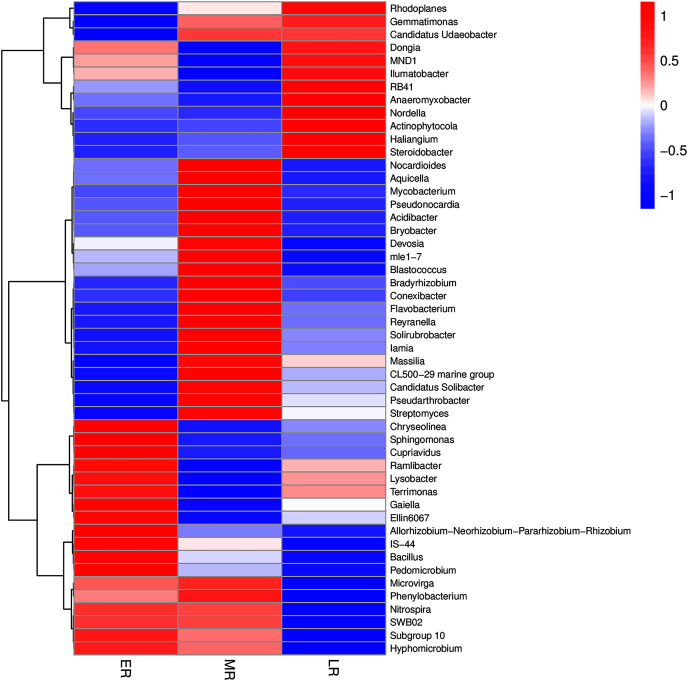
The heatmap of the top 50 genera in rhizosphere microbiota among three natural restoration stages. ER, Early restoration stage; MR, Middle restoration stage; LR, Later restoration stage.

**Figure 7:**
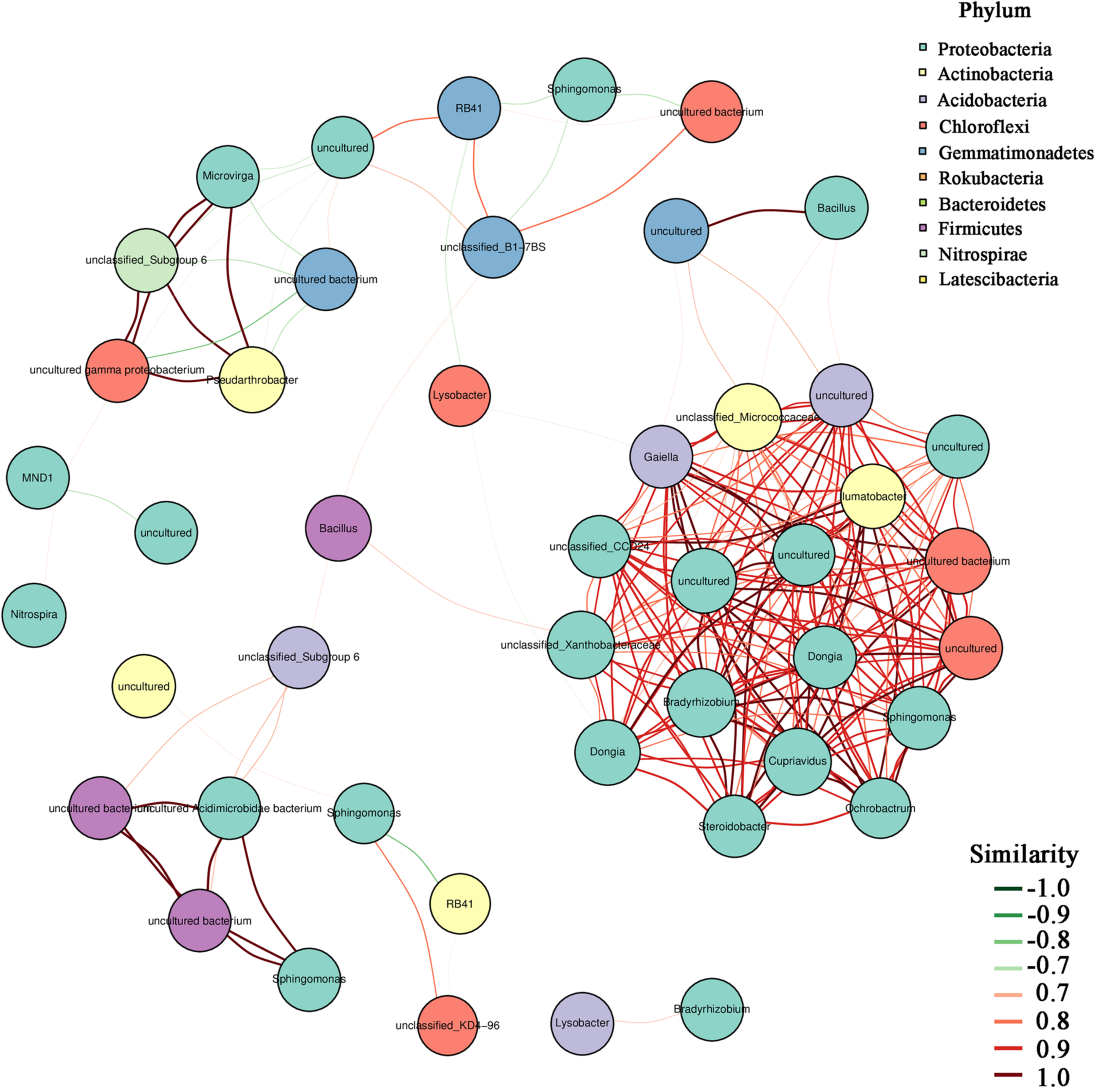
The dominant seed network of top 50 genera.

In addition, the heatmap of the top 50 domain genera in average abundance indicated that most genera among three natural restoration stages had obvious differences (Fig. 6). The dominant seed network figure showed that the *Bacillus* existed as a node genus in the 50 dominant seed networks among three natural restoration stages (Fig. 7).

## Discussion

The soil pollution caused by Sb mining in Xikuangshan is from Sb, As, Pb, Cd, Hg, Zn, and other heavy metals. Our investigation confirmed that the content of heavy metals in rhizosphere soil decreased significantly after long-term natural ecological restoration. Another study on the Xikuangshan mine also demonstrated that vegetation restoration is conducive to the reduction of soil heavy metal content (Guo et al., 2019). One possible explanation is that some plants with heavy metal tolerance and enrichment ability are retained during natural recovery, and the reduction of heavy metals in rhizosphere may be due to plant absorption.

Rhizosphere bacteria can support natural vegetation restoration by regulating biogeochemical processes, which can further help to improve the physical and chemical properties of rhizosphere soil (Mishra, Singh & Arora., 2017). Our present study observed that the beta diversity index of rhizosphere microbial community varied with different restoration stages, Other studies have also observed that the establishment and development of plant communities could alter rhizosphere bacterial beta diversity (Sun et al., 2018; Shrestha, Gautam & Ashwath, 2019). Different from the change of beta diversity, the alpha diversity (the Chao 1 index, Simpson index, Shannon index and Pielou index) under different restoration stages had various changes. Natural recovery significantly increased the Chao 1 index, but had no significant effect on the Simpson index, Shannon index and Pielou index, indicating that the main impact of natural restoration on rhizosphere bacteria was the number of rhizosphere microbes, not abundance or evenness. The alteration of rhizosphere bacterial diversity may be related to the change in plant species and rhizosphere soil habitats (Guo et al., 2019; Shrestha, Gautam & Ashwath, 2019).

At the phyla level, the ten dominant bacterial populations mainly included *Actinobacteria*, *Proteobacteria*, *Acidobacteri*a, *Chloroflexi*, *Gemmatimonadetes*, *Bacteroidetes*, *Rokubacteria*, *Firmicutes*, *Patescibacteria*, and *Planctomycetes*, indicating that these bacterial communities had strong adaptability and played an important role in three natural restoration stages (ER, MR, and LR) with Sb contaminated soil. *Proteobacteria*, the most abundant phyla in our study, exhibited significant survival and reproduction ability in mining area. This result is consistent with the researches in zinc mines (Luo et al., 2018) and copper mines (Liu et al., 2014; Sun et al., 2018), but not in gold mines where the predominant phyla is *Acidobacteria* (Sibanda et al., 2019). In this study, natural vegetation restoration led to a significant decrease in the relative abundance of *Patescibacteria* in the LR, and an increase in the relative abundance of *Actinobacteria*. These results were somewhat inconsistent with the observation that vegetation reconstruction in an iron mine could lead to the increases of *Patescibacteria* and the decreases of *Actinobacteria* (Deng et al., 2020). The reasons for this distinction may be attributed to the differences of vegetation and mining types.

Additionally, three phyla (*Calditrichaeota*, *Deferribacteres*, and *Epsilonbacteraeota*) were only found in ER. *Calditrichaeota* is an independent phylum recently recognized, and it is an anaerobic bacteria with oxygen tolerance and protein fermentation (Marshall et al., 2017). *Deferribacteres* mainly exists in the rhizosphere of specific restoration plant, such as *Phragmites karka* and *Typha latifolia* (Singh & Singh, 2018). In addition, *Deferribacteres*, often occurring in refinery wastes, can be used for *in-situ* bioremediation in polluted environments (Sarkar et al., 2016). *Epsilonbacteraeota*, a new microbial phyla in 2017, has a strong adversity tolerance, and mostly occurs in deep-sea hydrothermal vents and urban sewage (Wang et al., 2020). In general, the bacteria of these three phyla have high stress tolerances and certain preference and capacity to exist in a polluted environment, which can explain the reason that they only appeared in the heavily polluted rhizosphere soil at ER. These findings that *Calditrichaeota*, *Deferribacteres*, and *Epsilonbacteraeota* only appeared in ER, but not in MR and LR, which has an important indicator significance for soil heavy Sb contamination.

At the genus level, most of the dominant genera in rhizosphere during natural restoration stages are unclassified genera, which highlights the lack of knowledge about these less studied environments. The most abundant taxa in these environments included (1) *RB41*, belongs to *Acidobacteria* and exhibits a high sensitivity to soil fertility (Ai et al., 2018). *RB41* plays a key role in maintaining soil metabolism and biogeochemical function under long-term low nutrient stress conditions (Ai et al., 2018). *RB41* is the key genus in cadmium contaminated soil under saline alkali stress (Wang et al., 2019), and it is also the dominant genus in the petroleum-contaminated soil (Shen et al., 2018) and the coal mining areas (Sun et al., 2020). (2) *MND1* is also the dominant bacterial communities in the coal mining areas (Sun et al., 2020), the Weihe Terrace soil with lower concentrations of petroleum (Shen et al., 2018) and the whole soil shifts with crop growth (Hargreaves, Williams & Hofmockel, 2015). (3) *Ellin6067*, an ammonia-oxidizing bacteria (Xia et al., 2005), has a role in the degradation of xenobiotic and other complex organic compounds (Lezcano et al., 2017). The other unclassified genera included *IS-44*, *SWB02*, *Subgroup10*, *CL500-29_marine_group*, *Mle1-7* were found in rhizosphere soil, though they have a relative low relative abundance. For example, *SWB02*, a potential syntrophic bacteria, can establish magnetite-mediated direct electron transfer during the methanogenic degradation of volatile fatty acids (Lee et al., 2019). *CL500-29_marine_group*, an actinomycete, can effectively utilize a variety of carbon-based compounds (Lindh et al., 2015). Some of these unclassified taxa may originate from the original plants or air and insects and settle in rhizosphere soil through soil-root pathway. Further experiments are needed to better understand the succession of rhizosphere microbial community and their relationship between rhizosphere community and pollutant dynamics.

Also, some dominant bacteria in different genera showed a diversity change in the natural recovery process. *RB41* had the highest relative abundance in Sb mining, and this result was similar to Sun et al. (2018), which found that *RB41* was a dominant genus in copper mine tailings in Central China. These studies indicated that *RB41* could play an important role in maintaining soil metabolism and biogeochemical functions under long-term high pollution stress. From ER-MR-LR, the relative abundance of *RB41* and *Haliangium* increased significantly. *RB41* is the key genus in cadmium contaminated soil under saline alkali stress (Wang et al., 2019), and it is also the dominant genus in petroleum-contaminated soil (Shen et al., 2018). *RB41* has a high sensitivity to soil fertility (Ai et al., 2018). With the natural restoration, compound pollution of heavy metals decreased, and soil fertility might be gradually enhanced. The improved living environment can increase significantly the abundance of *RB41*.

*Haliangium* is a genus of bacteria from the family of *Kofleriaceae*. It was found to significantly increase in the rhizosphere soil of continuous cropping strawberry and mango (Li et al., 2019). As far as we know, this is the first time found that natural recovery could lead to a significant increase of *Haliangium* in Sb mining area, and the reasons need to be further studied.

In addition, *Bacillus* decreased significantly from ER-MR-LR. *Bacillus* has a strong tolerance to heavy metals, and can effectively reduce the absorption of heavy metals by plants through bioaccumulation and bio-transformation involving redox reactions (Ndeddy Aka & Babalola, 2016). Ramírez et al. (2019) have confirmed that the tolerance of *Bacillus* sp. MH778713 to Cr (VI) and Al can reach 15,000 mg/L and 10,000 mg/L, respectively. *Bacillus megaterium* H3 has the function of reducing Cr (VI) to Cr (III) with low toxicity, so as to reduce the harm of Cr to plants (Wang et al., 2018). Furthermore, *Bacillus thuringiensis* X30 can decrease the toxicity of Cu and Pb to plants and increase the biomass of crops (Han et al., 2018). Under Cd stress, *Bacillus* can promote rice growth, increase its biomass, photosynthetic pigment and micronutrient content, and reduce electrolyte permeability (Jan et al., 2019). Based on these results, we believe that *Bacillus* has a certain preference for heavy metal pollution, which can reduce the ecological toxicity of heavy metal and promote the growth of plants. Therefore, the *Bacillus* genus should be preferred in the early stage of vegetation restoration in Sb mining. In addition, our results also showed that *Bacillus* was a node genus in the dominant seed network, which had a great significance for maintaining the composition and diversity of rhizosphere microorganisms. With natural recovery, the changes in the composition of rhizosphere microorganisms were partly attributed to the significant decrease of *Bacillus*.

Also, with natural recovery, the abundance of *Gaiella* also showed a decreasing trend. *Gaiella* genus is sensitive to heavy metal pollution, and it is an important indicator bacteria in the local heavy metal remediation process (Luciana et al., 2011).

Furthermore, in this experiment, *Azorhizobium* was only detected in the ER phase. *Azorhizobium* can live in the plant rhizosphere in the lead-zinc mine tailings (Yang et al., 1997). Some *Azorhizobium* (*e.g., Azorhizobium caulinodans* ORS571) can colonize in plant roots and play a key role in nitrogen fixation in both symbiotic and aerobic free-living states (Ryu et al., 2020). Based on this, we speculate that *Rhizobium* genus has high application value in plant recovery in Sb mining for it not only can resist the high concentration of heavy metal, but also can improve the nitrogen fixation ability of plants in poor soil.

In sum, the abundance of rhizosphere bacteria were different in three natural restoration stages (ER, MR, and LR). *Calditrichaeota*, *Deferribacteres*, *Epsilonbacteraeota* phyla, and *Azorhizobium* genus only appeared in ER, and *Bacillus* and *Gaiella* genus had obviously high relative abundance in the ER. These rhizosphere bacteria had high tolerance and/or degradation ability to heavy metal pollution in Xikuangshan. The ways of bacteria tolerate and/or degrade heavy metals mainly included absorption, excretion, methylation, oxidation, and reduction of heavy metal, especially Sb (Filella, Belzile & Lett, 2007). Therefore, we can artificially add these rhizosphere bacteria to the rhizosphere of other plants, which will promote remediation of pollution soil in Sb contaminated soil. With natural recovery, the plant rhizosphere bacteria in the mining area reduced the toxic effects of heavy metal on plant roots through their accumulation and transformation (Mishra, Singh & Arora., 2017), resulting in the improvement of plant survival conditions and the change of vegetation types, and then changing the composition and abundance of rhizosphere bacteria (Deng et al., 2015). The change of rhizosphere microbial community could also influence the absorption of heavy metal by plants (Muehe et al., 2015).

## Conclusions

This is the first report on the diversity and composition of rhizosphere bacterial community in Sb mines with regard to the different natural restoration stages. Sequencing analysis of the rhizosphere soil showed that the alpha and beta diversity of rhizosphere bacteria were significantly different during three natural restoration stages (ER, MR, and LR). *RB41* and *Bacillus* genus had obviously high relative abundance in the ER. Three phyla (*Calditrichaeota*, *Deferribacteres*, and *Epsilonbacteraeota*) and *Azorhizobium* genus only appeared in the ER. These rhizosphere bacteria might be ideal bacteria for improving phytoremediation efficiency in the Sb mining region. In future research, we should try to isolate these rhizosphere bacteria with specific functions and explore their effects on phytoremediation alone or combination. This will provide an important basis for the combined remediation of mine contaminated soil by rhizosphere bacteria and plants.

## Supplemental Information

10.7717/peerj.12302/supp-1 Supplemental Information 1 Fig. S1.

10.7717/peerj.12302/supp-2 Supplemental Information 2 DNA sequence data and OTUs classified data of 15 composite rhizosphere soil samples among three natural restoration stages (ER, MR, and LR).

10.7717/peerj.12302/supp-3 Supplemental Information 3 Raw data of alpha diversity at three natural restoration stages.

10.7717/peerj.12302/supp-4 Supplemental Information 4 Raw data of the top ten bacteria phylum in abundance at three natural restoration stages.

10.7717/peerj.12302/supp-5 Supplemental Information 5 Raw data of the concentrations of heavy metals at three natural recovery stages.

## Additional Information and Declarations

### DNA Deposition

The following information was supplied regarding the deposition of DNA sequences: The sequences from rhizosphere bacterial samples are available at NCBI SRA: BioProject PRJNA743772.

### Data Availability

The following information was supplied regarding data availability: Data is available at NCBI SRA, BioSample accessions: SAMN20060126 to SAMN20060140.
