# Supplementary figures and images for: Changes in diversity and composition of rhizosphere bacterial community during natural restoration stages in antimony mine
Source: PeerJ. 2021 Oct 14;9:e12302. doi: 10.7717/peerj.12302 (PMC8520691; doi:10.7717/peerj.12302)

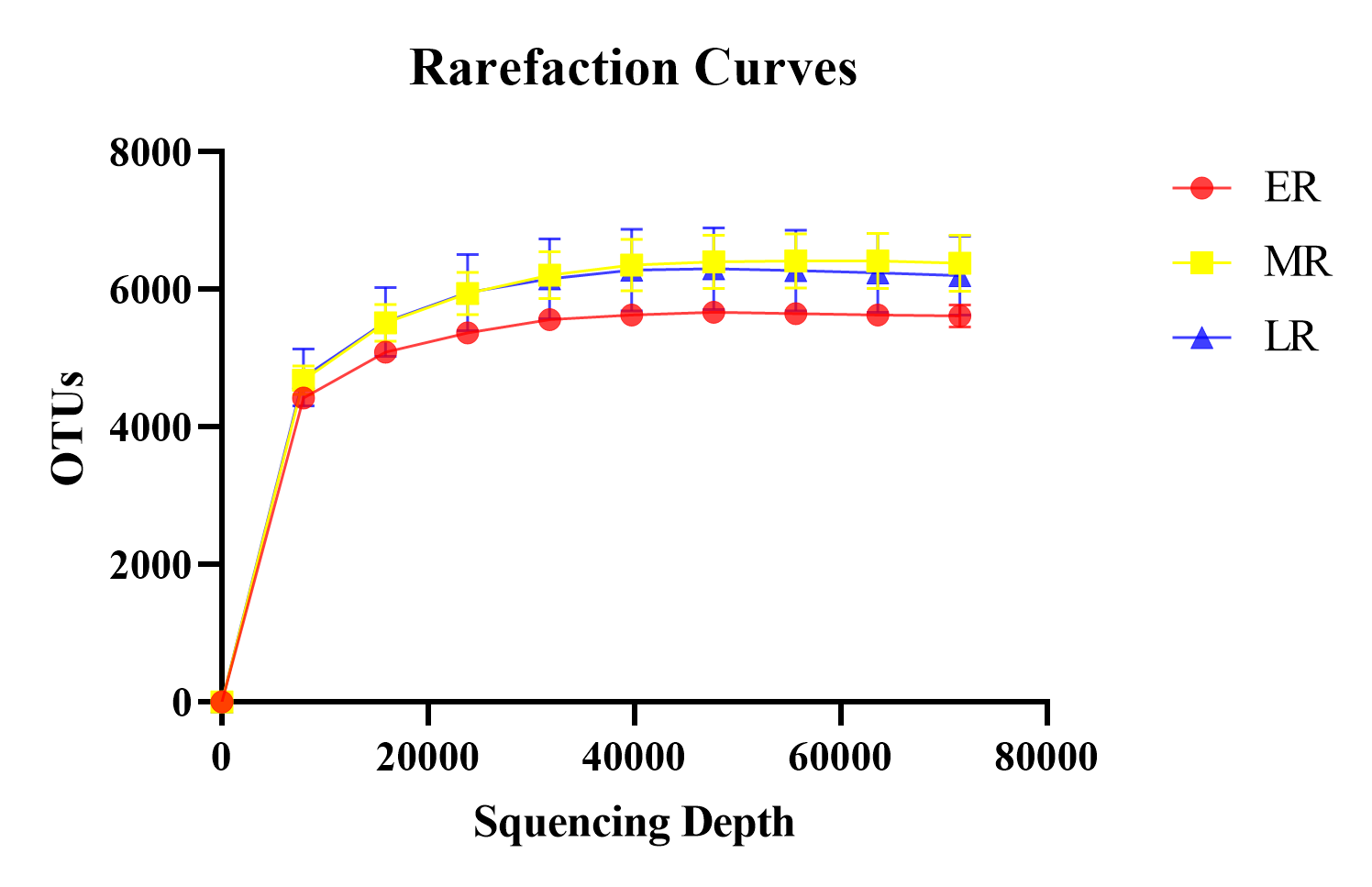

Supplement: Supplemental Information 1 [file peerj-09-12302-s001.png]
