# Supplementary material for: Changes in diversity and composition of rhizosphere bacterial community during natural restoration stages in antimony mine
Source: PeerJ. 2021 Oct 14;9:e12302. doi: 10.7717/peerj.12302 (PMC8520691; doi:10.7717/peerj.12302)
Supplement: Supplemental Information 2 [file peerj-09-12302-s002.doc]

**Table S1** DNA sequence data and OTUs classified data of 15 composite rhizosphere soil samples among three natural restoration stages (ER, MR, and LR).

| Sample ID | Raw reads | Effective reads | Phylum | Class | Order | Family | Genus | Species |
| --- | --- | --- | --- | --- | --- | --- | --- | --- |
| ER1 | 159373 | 142659 | 31 | 83 | 148 | 187 | 252 | 62 |
| ER2 | 203444 | 184021 | 28 | 85 | 161 | 207 | 317 | 91 |
| ER3 | 195100 | 174228 | 32 | 88 | 169 | 221 | 316 | 100 |
| ER4 | 145013 | 127839 | 33 | 89 | 163 | 203 | 285 | 71 |
| ER5 | 203861 | 181421 | 27 | 84 | 144 | 199 | 276 | 80 |
| MR1 | 230420 | 204509 | 30 | 86 | 158 | 221 | 329 | 130 |
| MR2 | 226715 | 201163 | 24 | 77 | 141 | 207 | 332 | 135 |
| MR3 | 305152 | 272162 | 28 | 80 | 151 | 196 | 293 | 102 |
| MR4 | 221322 | 195941 | 25 | 69 | 130 | 209 | 322 | 139 |
| MR5 | 228838 | 206606 | 27 | 77 | 146 | 208 | 316 | 104 |
| LR1 | 219664 | 197269 | 31 | 92 | 164 | 196 | 301 | 76 |
| LR2 | 219192 | 194296 | 24 | 82 | 139 | 191 | 270 | 84 |
| LR3 | 231687 | 204471 | 26 | 76 | 147 | 195 | 281 | 77 |
| LR4 | 198331 | 174337 | 25 | 82 | 147 | 196 | 293 | 89 |
| LR5 | 164588 | 148966 | 25 | 75 | 130 | 159 | 191 | 38 |
